# Variability of laying hen behaviour depending on the breed

**DOI:** 10.5713/ajas.18.0645

**Published:** 2019-01-02

**Authors:** Agnieszka Kozak, Kornel Kasperek, Grzegorz Zięba, Iwona Rozempolska-Rucińska

**Affiliations:** 1 Institute of Biological Basis of Animal Production, University of Life Sciences in Lublin, Lublin 20-950, Poland

**Keywords:** Laying Hen, Behavioural Test, Breed

## Abstract

**Objective:**

For many generations, most species of farm animals have been subjected to intense and strictly targeted selection for improvement of their performance traits. This has led to substantial changes in animal anatomy and physiology, which resulted in considerable differences between the current animal breeds and their wild ancestors. The aim of the study was to determine whether there is breed-specific variability in behaviour as well as differences in emotional reactivity and preferences of laying hens.

**Methods:**

The investigations involved 50 Green-legged Partridge, 50 Polbar, and 50 Leghorn hens. All birds were kept in the same conditions, and the behavioural tests were carried out at 30 weeks of age. We used the tonic immobility test and a modified open-field test including such objects as water, commercial feed, feed enriched with cereal grains, finely cut straw, and insect larvae, a sandpit, a mirror, and a shelter imitating a hen nest.

**Results:**

The research results demonstrate that the birds of the analysed breeds differ not only in the excitability and emotional reactivity but, importantly, also in the preferences for environment-enriching elements. Ensuring hens’ well-being should therefore be based on environmental modifications that will facilitate acquisition of essential elements of chickens’ behaviour. The greatest emotional reactivity was found in the Leghorn breed, which may be a result of correlated selection aimed at an increase in chicken productivity.

**Conclusion:**

The differences in the behaviour of the birds from the analysed breeds indicate that laying hens cannot be regarded as one group of animals with the same environmental requirements.

## INTRODUCTION

Most farm animal species have long been subjected to intense and strictly targeted selection for improvement of their performance traits. Programs implemented in chicken breeding are mainly focused on increasing weight gain, laying performance, or internal and external egg quality traits. This has led to substantial changes in the anatomy and physiology of this species, which resulted in considerable differences between birds reared currently and their wild ancestors, also in terms of their behaviour. Natural hens’ behaviour represents a repertoire of their ancestors’ behaviours, provided that their rearing conditions allow demonstration of such behaviours [1]. Behavioural problems arise when chickens are motivated to show certain behaviours but they are unable to express them due to limitations such as the size of the cage or absence of enrichment elements. Consequently, other variants of behaviour arise and can often lead to behavioural disorders, e.g. feather plucking. Behavioural patterns depend not only on birds’ habitat and experiences but also on the genetic background, environmental conditions prevailing during embryonic development, and epigenetic effects [2,3]. Therefore, it seems that each breed of laying hens reared on farms can exhibit diverse behavioural needs for maintenance of homeostasis of the organism. However, the differences in behavioural needs between chicken genotypes are not considered during adjustment of rearing standards to ensure bird welfare. Therefore, standardisation of the conditions for rearing laying hens on farms does not necessarily improve the welfare of all breeds.

The aim of the study was to determine whether there is variability in behaviour as well as differences in emotional reactivity and preferences of laying hens depending on the breed.

## MATERIALS AND METHODS

### Birds and husbandry

All procedures employed during the research were approved by the II Local Ethics Committee for Animal Testing at the University of Life Sciences in Lublin, Poland (Approval No. 69/2017 of 28 September 2017). The investigations involved 150 birds, including 50 Green-legged Partridge (Zk), 50 Polbar (Pb), and 50 Leghorn (LG) hens. The Green-legged Partridge is a native chicken breed described at the end of the 19th century. It is often reared on organic farms [4]. The hens are perfectly adapted to the conditions of extensive rearing in free-range systems. Polbar is a Polish autosexing synthetic breed produced by mating Green-legged Partridge hens with Plymouth Rock cocks. The Polbar breed traits were conserved in the 1950s. It should be noted that both breeds (Zk and Pb) are reared in closed, unselected populations in accordance with the phenotypic and functional pattern developed for protection of animal genetic resources [5]. The Leghorn is one of the most popular laying hen breeds in Europe, which is especially adapted to intensive breeding and subjected to intensive selection towards performance traits [6].

All birds were kept in the same farm building on straw bedding, which guaranteed identical rearing conditions. The hens were 30 weeks old at the time of the experiment. The birds were kept in 6 group boxes, with 25 females of one line in each at a density of 0.3 m^2^/bird. All boxes were equipped with nipple drinkers, feeders, and nests, and with 16 hours of light each day. The birds were fed and maintained according to standard breeding requirements for poultry. The tests were carried out from 8.00 to 15.00 h for 6 days (1 day = 1 box).

### Open-field test

All birds were subjected to the open-field test individually [7] for 10 min. The test was carried out in an observation box with a 1.25×1.25 m floor divided into 25 squares with an area of 25×25 cm each. A camera viewing the entire area available for the birds was mounted above the box and each test was recorded. The open-field test was modified, i.e. additional elements enriching the environment were placed in the box. These elements included a container with water, a container with commercial feed, a container with feed supplemented with cereal grains, finely cut straw, and insect larvae, a sandpit, a mirror, and a shelter imitating hen nests. The birds were placed in the central point of the box to keep the same distance from the enriching objects. The analysis of film recordings was carried out to determine the duration of animal’s exploration of the objects and locomotion throughout the test measured by means of a hand-held stopwatch. Such behaviours as vocalisation, defecation, comfort behaviour, interest in the floor, and shake-off were noted. The analysed elements and the measurement procedure are presented in Table 1.

### Tonic immobility

Immediately after the open-field test, the tonic immobility test described by Jones [8] was applied. The birds were immobilised by being placed in a special cradle [6] on the back. Their sternum was pressed gently in such a position that the head could hang backwards freely. The latency of tonic immobility was measured. Upon the tonic immobility, the experimenter released the pressure and latency to the first head movement and straightening (commonly referred to as the duration of tonic immobility test [TI1]) was measured (tonic immobility 2 [TI2]) [8].

### Statistical analysis

Since the recorded traits do not have a normal distribution, the data were rank-transformed [9] Bonferroni-corrected multiple comparisons of the estimations of differences in the analysed traits between the breeds were analysed with 2-factor models considering the effect of the genetic group and the object of interest. The analyses were performed with the use of the GLIMMIX procedure in the SAS version 9.4 program (SAS Institute, Cary, NC, USA). The probability of the occurrence of a specific reaction depending on the bird breed was determined as well. The significance of the differences was verified by the analysis of variance and the least square method considering the fixed effect of the genetic group in the model (SAS Institute, USA).

The results are presented as arithmetic means (Tables 2, 3) to facilitate biological interpretation.

## RESULTS

### Open field test and tonic immobility

An almost two-fold longer TI1 was noted in the LG hens than in the Zk and Pb breeds (Table 2) and the differences were statistically significant (Table 4). The TI2 was almost three-fold shorter in Pb than in Zk, with statistical significance of the differences (Table 4). There were no statistically significant differences in the latency of undertaking activity and exploration and in the total duration of locomotion and examination of the objects between the tested chicken breeds (Table 4). However, the number of squares covered by the LG hens was several-fold greater than that in the Pb and Zk breeds.

### Defecation and vocalisation

A highly significant difference was observed in the probability of occurrence of defecation, self-grooming behaviour, and shake-off behaviour between the LG hens and the Zk and Pb hens. The probability of vocalisation was several-fold higher in Pb and LG than in Zk (Table 5).

### Elements enriching the environment

The Pb birds showed the greatest interest in the water, as they spent nearly twice as much time at the drinker than the Zk and LG hens; however, these differences were not statistically significant (Tables 3, 6). The longest time spent on eating the commercial feed was exhibited by the LG chickens. In turn, the greatest interest in the enriched feed was noted in the case of the Zk, which also exhibited the greatest interest in this object (Table 3). The shelter was an enrichment element that received the lowest interest from the Pb hens, and the differences were statistically significant in comparison with the other breeds (Tables 3, 6). The mirror aroused the greatest interest among the LG birds, which devoted over a 5-fold and 3-fold longer time than the Zk and Pb breeds, respectively (Table 3). There were no differences between the genetic groups in the time devoted to examination of the floor and in the interest in the sandpit (Table 6).

## DISCUSSION

A major hen welfare-related problem in modern industrial poultry farming is the lack of adjustment of rearing conditions to birds’ behavioural needs [11]. The natural behaviours of domestic fowl, e.g. free movement, pecking, scratching the ground, wing flapping, self-grooming, or quiet rest and sleep, may be limited by the impossibility to express them. The tests carried out in this study were aimed at verification whether the many-generation hen selection, which indirectly determines birds’ behaviour [12], exerted an effect on the behavioural variability and whether birds from different breeds characterised by dissimilar performance value exhibited different behaviours.

The results demonstrated such differences between the analysed breeds. Noteworthy is the relatively long time devoted by the Zk birds to explore the enriched feed; this parameter had several-fold higher values than in the case of the Pb and LG breeds. The birds did not only ingest the feed but also expressed the need for scratching and searching, thus satisfying one of the basic needs, i.e. curiosity [13]. However, it seems that the scratching and searching need is strongly developed mainly in the Zk birds, which represent primitive breeds that have not been selected towards high performance value. This element of the environment was not preferred by the hens from the other breeds. The LG hens, which are mainly reared as high-yield layers in intensive production, exhibited considerably greater interest in the commercial feed, which has a form of a homogeneous granulate and ingestion thereof does not require searching for edible parts. These results indicate that selection can significantly change hens’ behaviour and food preferences. High-yield breeds are targeted at feed intake that will fulfil their physiological rather than behavioural needs. For economic reasons, bird breeds with the highest feed conversion rates and absence of the scratching and searching behaviour are preferred in breeding.

Another enriching element, i.e. the mirror, showed considerable differences in curiosity among the breeds. The longest time for examination of the object was devoted by the LG hens. The birds pecked at their reflection in the mirror. The interest in the mirror may indicate great curiosity in this breed. It should be noted that this temperament feature cannot be fully satisfied in the farm rearing conditions. A stimulus-poor and monotonous environment does not offer opportunities to satisfy curiosity. Hence, it is possible that boredom and an attempt to satisfy curiosity is one of the causes of feather plucking, which is quite a common phenomenon in the LG breed (own unpublished observations).

There were also differences in the birds’ interest in the shelter. This object was clearly avoided by the Pb breed. Simultaneously, none of the enriching elements was found to define the preferences of this breed, as in the case of Zk and LG. This may be a result of the origin and the components of this breed, i.e. heavy meat breeds and the primitive Zk breed. Hence, the behaviour of these hens is characterised by elements differing them from light layer breeds such as the LG and from the Zk breed. It can therefore be concluded that hybrids of layer breeds reared in a farm breeding system will differ in their preferences and behaviour from parent breeds. This is an important finding, as it indicates that the assessment of chicken behaviour cannot be limited to testing pure-bred birds.

There were differences in the behaviour of the analysed hen breeds in terms of emotional reactivity. Animals respond adequately to the degree of emotional arousal, which is strongly associated with the breed in addition to individual traits. The indicators of the emotional status comprise the locomotion speed, vocalisation, defecation, and self-grooming. The present investigations demonstrated differences in the level of these indicators depending on the breed. Within a similar locomotion time (no statistical differences), the LG hens covered a several-fold higher number of squares on the floor, which evidenced a fast locomotion rate. Additionally, there was a significantly greater probability of defecation in this breed. These indicators might suggest increased anxiety and higher fearfulness in LGs [14]; however, another group of behaviours associated with comfort activities (e.g. cleaning feathers, flapping wings, ruffling feather, scratching the body) was noted in this breed more often than in the others (Table 5). As reported by Zimmerman et al [14] increased locomotion is correlated with anxiety about an upcoming aversive event, whereas anticipation of a positive event is associated with comfort behaviours (e.g. cleaning feathers, flapping wings, ruffling feather, scratching the body). It should be underlined that application of a standard open-field test for assessment of fearfulness [15,16] without modifications consisting in enrichment of the environment with additional elements would suggest a high level of fear in the LG hens. In this case, however, the presence of the interest in the elements of the environment with the absence of significant differences between the investigated breeds should be emphasised. Simultaneously, if the animal shows interest in objects, increased locomotion should not be regarded as expression of stress, as a stressed and terrified animal does not explore the environment. The relationships between the emotional reaction to the environment and the decision to avoid or approach the environment are key elements of animal welfare [17]. The behaviour of the LG hens indicates that the breed is characterised by very strong emotional arousal and high reactivity, but not necessarily fearfulness. The present results agree with investigations demonstrating that birds with white plumage exhibit higher emotional reactivity than birds with coloured feathers [18–20]. There were no differences in the level of reactivity between the coloured Zk and Pb breeds. This result should not be surprising, given the origin of the Pb breed. Importantly, only the LG breed has been intensively selected for many generations towards higher laying performance and it is currently characterised by very high laying rates. The Zk and Pb hens belong to conservative herds and constitute a genetic reserve; therefore, no selection targeted at birds’ performance traits is being carried out. In conclusion, the higher reactivity of LG hens may also be a result of indirect selection and a behavioural response to the high physiological requirements of the organism.

The present investigations also included a tonic immobility test, whose results can be an indicator of the level of fearfulness, as suggested by various researchers [8,21]. Tonic immobility is an unconditioned reaction that can be easily induced manually and is reflected by bird’s immobility and lower reactivity to external stimulation [9]. It is believed that the latency of the first head movement and the TI duration are positively correlated with fearfulness [22]. In the present study, the shortest duration of immobility was recorded in the Pb breed; however, the results of the open-field test do not show lower fearfulness in this breed in comparison to the others. There were no differences between the breeds in the time required by the birds to start exploration of the environment or in the locomotion time, which can undoubtedly be indicators of the level of fear [10]. TI is thought to be a behaviour protecting birds from predators [23,24]. Given this theory, it can be assumed that primitive behaviours related to species survival can be eliminated by selection. The LGs needed twice as long latency of tonic immobility, which did not influence the duration of immobility. Possibly, selection eliminates the physiological reaction of immobility but not its duration. The duration of immobility was the longest in the primitive Zk breed; yet, statistically significant differences were noted exclusively between Zk and Pb, and not between the specialised LG breed. As indicated by the other research results, tonic immobility is difficult to achieve by excitable birds with increased emotional reactivity. Thus, the latency of immobility may be a specific marker of emotional excitability of an individual.

## CONCLUSION

To sum the study results, it can be concluded that the birds from the different breeds differ not only in the degree of excitation and emotional reactivity but, importantly, also in their preferences for environment-enriching elements. Ensuring well-being should therefore consider environmental modifications that will facilitate expression of characteristic and essential elements of the breed behaviour. The greatest emotional reactivity was detected in the LG breed, which may be a result of selection targeted at increasing chicken productivity [20]. The differences in the behaviour of birds from the analysed breeds indicate that laying hens cannot be regarded as one group of animals with the same environmental requirements. Therefore, establishment of welfare requirements should be based on verification of elements that are indeed essential for a particular genetic line to prevent disturbance in the homeostasis of the organism and to limit the occurrence of behavioural abnormalities.

## Figures and Tables

**Table 1:** Assessment of hen behaviour during the enriched open-field test

Object/Behaviour	Measurement procedure
Latency of undertaking activity	Time between placing the bird on the floor of the experimental box and the first movement, e.g. Head movements, looking around, but no locomotor activity (s)
Latency of undertaking exploration	Time between placing the bird on the floor in the experimental box and the beginning of locomotor activity (s)
Time of locomotion	Time spent by the hen on locomotion throughout the test, excluding the activity time;measured in seconds (s)
Time of exploration of objects	Time spent by the hen observing and showing interest in the objects; measured in seconds (s)
Exploration area	Number of squares covered by the tested hen
Water (W)	Time spent on exploration of the object or using its resources; measured in seconds (s)
Commercial feed (CF)	Time spent on exploration of the object or using its resources; measured in seconds (s)
Enriched feed (EF)	Time spent on exploration of the object or using its resources; measured in seconds (s)
Sandpit (SA)	Time spent on exploration of the object or using its resources; measured in seconds (s)
Mirror (M)	Time spent on exploration of the object or pecking at own reflectionin the mirror; measured in seconds (s)
Shelter (S)	Time spent on exploration of the object or staying inside; measured in seconds (s)
Floor (F)	Time spent on exploration of the floor; measured in seconds (s)
Vocalisation	Assessed in a 0–1 point scale, where 0 denoted no vocalisation, 1-presence of vocalisation
Defecation	Assessed in a 0–1 point scale, where 0 denoted no defecation, 1 - presence of defecation
Comfort behaviour	Assessed in a 0–1 point scale, where 0 denoted no comfort behaviour, 1 - presence of comfort behaviour
Shake-off	Assessed in a 0–1 point scale, where 0 denoted no shake-off, 1 - presence of shake-off

**Table 2:** Mean level of traits defining the emotional reactivity of birds depending on the breed

Breed	Variable	Mean	Standard error	Min	Max
Leghorn	Latency of tonic immobility	60.4	59.8	0	322
	Duration of tonic immobility	28.5	52.4	0	278
	Latency of activity	81.6	137.9	1	600
	Latency of exploration	284.5	230.3	2	600
	Time of examination of objects	518.4	137.9	0	599
	Duration of locomotion	316.4	229.7	0	598
	Explored area	25.2	33.9	0	110
Polbar	Latency of tonic immobility	39.3	52.2	0	247
	Duration of tonic immobility	11.7	24.9	0	111
	Latency of activity	92.0	197.9	1	600
	Latency of exploration	257.0	237.8	1	600
	Time of examination of objects	508.0	197.9	0	599
	Duration of locomotion	343.0	237.8	0	599
	Explored area	6.0	8.7	0	44
Green-legged Partridge	Latency of tonic immobility	35.0	27.7	0	111
	Duration of tonic immobility	36.2	53.0	0	217
	Latency of activity	47.5	141.8	1	600
	Latency of exploration	303.3	244.4	1	600
	Time of examination of objects	552.5	141.8	0	599
	Duration of locomotion	297.5	244.3	0	599
	Explored area	5.2	8.1	0	44

**Table 3:** Mean duration of interest in the objects depending on the breed

Breed	Object	Mean	Standard error	Min	Max
Leghorn	Water (W)	26.8	65.1	0	276
	Commercial feed (CF)	49.3	100.4	0	514
	Enriched feed (EF)	5.9	17.3	0	106
	Shelter (S)	13.3	34.5	0	193
	Mirror (M)	47.1	66.0	0	232
	Sandpit (SA)	21.6	65.6	0	429
	Floor (F)	4.2	18.1	0	120
Polbar	Water (W)	55.7	112.8	0	464
	Commercial feed (CF)	6.5	18.6	0	92
	Enriched feed (EF)	15.4	41.0	0	241
	Shelter (S)	1.8	8.1	0	53
	Mirror (M)	18.9	55.9	0	349
	Sandpit (SA)	65.0	114.2	0	508
	Floor (F)	43.5	67.9	0	241
Green-legged Partridge	Water (W)	25.7	62.7	0	340
	Commercial feed (CF)	18.3	59.2	0	355
	Enriched feed (EF)	59.6	119.2	0	561
	Shelter (S)	11.2	30.6	0	172
	Mirror (M)	9.0	32.0	0	146
	Sandpit (SA)	32.5	74.4	0	411
	Floor (F)	8.8	24.3	0	126

**Table 4:** Estimators of differences in traits defining the emotional reactivity of birds depending on the breed, their significance and confidence interval

Type of test	Trait (unit of measurement)	Breed		Estimate	Pr> |t|	Adjust lower	Adjust upper
Tonic immobility	Latency of tonic immobility (s)	LG	Pb	0.42	0.031	−0.046	0.887
		LG	Zk	0.52	0.007	0.057	0.991
		Pb	Zk	0.10	0.592	−0.363	0.570
	Duration of tonic immobility (s)	LG	Pb	0.35	0.067	−0.110	0.818
		LG	Zk	−0.17	0.381	−0.632	0.296
		Pb	Zk	−0.52	0.007	−0.986	−0.058
Open field test	Latency of activity (s)	LG	Pb	0.13	0.489	−0.332	0.598
		LG	Zk	0.28	0.154	−0.190	0.740
		Pb	Zk	0.14	0.461	−0.323	0.607
	Latency of exploration (s)	LG	Pb	0.07	0.703	−0.348	0.478
		LG	Zk	−0.12	0.468	−0.537	0.289
		Pb	Zk	−0.19	0.269	−0.602	0.224
	Explored area	LG	Pb	0.68	0.000	0.231	1.130
		LG	Zk	0.76	0.000	0.312	1.210
		Pb	Zk	0.08	0.666	−0.369	0.530
	Duration of examination of objects (s)	LG	Pb	−0.32	0.091	−0.774	0.136
		LG	Zk	−0.07	0.698	−0.528	0.382
		Pb	Zk	0.25	0.192	−0.209	0.701
	Duration of locomotion (s)	LG	Pb	−0.27	0.165	−0.748	0.201
		LG	Zk	−0.06	0.743	−0.539	0.410
		Pb	Zk	0.21	0.288	−0.266	0.684

LG, Leghorn; Pb, Polbar; Zk, Green-legged Partridge.

**Table 5:** Probability of occurrence of reactions depending on the breed

Type of test	Trait (unit of measurement)	Zk		Pb		LG	
		
		LSM	SE	LSM	SE	LSM	SE
Open field test	Probability of occurrence of defecation	0.18^B^	0.04	0.08^B^	0.04	0.8^A^	0.05
	Probability of occurrence of comfort behaviours	0.22^B^	0.06	0.32^B^	0.06	0.62^A^	0.06
	Probability of occurrence of shake-off behaviour	0.06^B^	0.04	0.18^B^	0.04	0.6^A^	0.05
	Probability of occurrence of vocalisation	0.08^A^	0.05	0.34^B^	0.05	0.36^B^	0.05

Zk, Green-legged Partridge; Pb, Polbar; LG, Leghorn; LSM, lease squares means; SE, standard error.

A,B Means marked with different letters differ significantly between the breeds at p≤0.001.

**Table 6:** Estimators of differences in traits defining birds’ interest in the object depending on the breed, their significance and confidence interval

Object	Breed	Breed	Estimate	Probability	Adjust lower	Adjust upper
Water (W)	LG	Pb	−38.16	0.373	−196.12	119.80
	LG	Zk	5.41	0.899	−152.54	163.36
	Pb	Zk	43.57	0.309	−114.44	201.58
Commercial feed (CF)	LG	Pb	221.31	0.000	63.35	379.27
	LG	Zk	171.29	0.000	13.34	329.24
	Pb	Zk	−50.02	0.243	−208.03	107.99
Enriched feed (EF)	LG	Pb	21.17	0.621	−136.79	179.13
	LG	Zk	−97.85	0.023	−255.80	60.10
	Pb	Zk	−119.02	0.006	−277.03	38.99
Shelter (S)	LG	Pb	126.55	0.003	−31.41	284.51
	LG	Zk	43.22	0.313	−114.73	201.17
	Pb	Zk	−83.33	0.050	−241.34	74.68
Mirror (M)	LG	Pb	163.93	0.000	5.97	321.89
	LG	Zk	198.47	0.000	40.52	356.42
	Pb	Zk	34.54	0.420	−123.47	192.55
Sandpit (SA)	LG	Pb	−68.27	0.111	−226.23	89.69
	LG	Zk	−1.76	0.967	−159.71	156.19
	Pb	Zk	66.51	0.121	−91.50	224.52
Floor (F)	LG	Pb	−13.58	0.751	−171.54	144.38
	LG	Zk	104.49	1.000	−53.46	262.44
	Pb	Zk	118.07	0.769	−39.94	276.08

LG, Leghorn; Pb, Polbar; Zk, Green-legged Partridge.
